# Clinical study on the selection of endoscopes and microscopes for transsphenoidal surgery of non-aggressive pituitary macroadenoma and microadenoma and the influencing factors of hyposmia after endoscopic transsphenoidal surgery

**DOI:** 10.3389/fneur.2024.1321099

**Published:** 2024-02-29

**Authors:** Fanyi Kong, Weiping Cheng, Qingyang Zhan

**Affiliations:** ^1^First Affiliated Hospital of Harbin Medical University, Harbin, China; ^2^First Affiliated Hospital of Heilongjiang University of Chinese Medicine, Harbin, China; ^3^Department of Neuroscience, Institute of Chinese Medicine, Heilongjiang University of Chinese Medicine, Harbin, China

**Keywords:** pituitary adenomas, neuroendoscope, microscope, curative effect comparison, transsphenoidal surgery

## Abstract

**Background and objective:**

Transsphenoidal surgery, including endoscopic and microscopic resection, is the first choice of treatment for pituitary tumors. With the widespread application of neuroendoscopy in recent decades, there has been a trend to replace microscopes. In clinical practice, we have found that in transsphenoidal surgery for non-invasive microadenomas and macroadenomas, microscopy can achieve a higher total resection rate, shorter operation time, lower incidence of postoperative complications, and faster recovery of olfaction. This study aimed to explore the selection of endoscopes and microscopes for non-aggressive transsphenoidal surgery for pituitary adenomas and the factors affecting olfactory recovery.

**Methods:**

From August 2019 to October 2022, 93 patients with non-aggressive microadenomas and macroadenomas via the transsphenoidal approach were selected from the First Affiliated Hospital of Harbin Medical University and treated with rich experience in pituitary tumor subspecialty microscopy and endoscopic surgery. Different surgical methods were used to divide the patients into microscopic (*n* = 35) and endoscopic (*n* = 58) groups. The total tumor removal rate, intraoperative blood loss, operation time and cost, postoperative hospital stay, recovery of visual function, postoperative changes in hormone levels, complication rate, and recovery from complications 3 months after the operation were compared between the two groups.

**Results:**

There were no significant differences in the tumor removal rate, postoperative visual acuity, and visual field recovery between the two groups (*p* > 0.05). There was a significant difference in the recovery rate of olfactory function between the two groups 3 months after the operation (*p* < 0.05), and there was no significant difference in the incidence of other complications (*p* > 0.05); Compared with the two groups, the microscope group had shorter operation time, longer postoperative hospital stay, less average operation cost and less blood loss, and the difference was statistically significant (*p* < 0.05). The position of the nasal septum mucosal flap incision was a risk factor for hyposmia 3 months after the operation.

**Conclusion:**

Microsurgery and endoscopic surgery are suitable surgical treatments for nonaggressive microadenomas and macroadenomas. The total tumor removal and postoperative hormone remission rates of the two surgical methods were approximately the same. However, the microsurgery group had a shorter operation time, less intraoperative blood loss, faster olfactory function recovery, and a lower average operation cost. The position of the nasal septal mucosal flap incision was a risk factor for hyposmia at 3 months postoperatively. Hyposmia is less likely to occur when the superior edge of the nasal septal mucosal flap incision is not higher than the lower edge of the ipsilateral superior turbinate.

## 1 Introduction

Pituitary adenoma is a common benign endocrine tumors that is often accompanied by visual field damage and deterioration (1). The clinical symptoms of pituitary adenoma include visual field defects, headaches, abnormal hormone secretion, and other symptoms caused by tumor compression of the pituitary gland and surrounding tissues, which seriously affect the quality of life of patients. Transsphenoidal surgery remains the first choice for treating pituitary adenomas, with transsphenoidal microsurgery and transsphenoidal endoscopic surgery being the two main surgical methods. With the innovation and development of science and technology, and the continuous improvement of neurosurgical microscopy and endoscopic technology, these minimally invasive surgical methods are becoming increasingly mature, reducing postoperative complications, reducing intraoperative bleeding, and improving patients’ survival rate and quality of life. Although both minimally invasive methods have advantages and claims, there is a need for a clear consensus on whether they are superior. However, with the wide application of neuroendoscopy in recent decades, it has been favored by neurosurgeons for its close observation, precise observation of tumors, pituitary tissue, and surrounding tissue structure, expansion of the visual space of the surgical field, and arbitrary selection of the ideal surgical angle. There is a trend to replace the use of microscopes.

Some studies (2, 3) have compared endoscopic transsphenoidal surgery with conventional microscopic surgery. Most studies (4–6) have shown that endoscopic surgery for giant pituitary adenomas, incredibly invasive pituitary adenomas, is more effective, with fewer complications and recurrences, and is conducive to the recovery of visual function and hormone levels. With the improvement in patients’ quality of life after surgery, a series of symptoms caused by nasal destruction during endoscopic transsphenoidal surgery has become the focus of attention in various studies (7–12). The removal rate and postoperative complications of microscopic and endoscopic resection of pituitary adenomas are closely related to the level of operation and the proficiency of the operator. The actual situation of every single center is also limited by various factors, and the size of the study sample will also affect the conclusion; therefore, different research institutions draw different conclusions (9, 13, 14).

In clinical practice, we found that in transsphenoidal surgery for non-invasive microadenomas and macroadenomas, the operation under the microscope can be curetted to the tumor boundary to achieve a higher total resection rate, shorter operation time, and lower incidence of postoperative complications. Therefore, we performed a comparative endoscopic and microscopic study of these tumors.

## 2 Materials and methods

### 2.1 Data of clinical

#### 2.1.1 Experimental plan

In this single-center retrospective comparative study conducted at the First Affiliated Hospital of Harbin Medical University between June 2019 and October 2022, 93 patients diagnosed with pituitary microadenoma and macroadenoma were included. Transnasal transsphenoidal pituitary adenoma resection was performed on all patients by the same surgeon and three assistants experienced in endoscopic and microscopic surgery for pituitary adenomas. Initially, the effect of individual differences on the interpretation of the results was ignored. Patients meeting surgical indications between June 19, 2019, and June 21, 2021, underwent microsurgery, while those from June 21, 2021, to October 22, 2022, received endoscopic treatment. Complete medical records, preoperative and postoperative imaging data, visual field examination results, and hormone level examinations were available for all patients, who were divided into neuroendoscopic and microscopic groups according to the surgical method. Postoperative monitoring was conducted, and follow-up occurred 3 months later through outpatient visits or phone calls, with two datasets organized for statistical analysis and comparison.

#### 2.1.2 Inclusion criteria

Inclusion criteria involved patients who underwent transsphenoidal surgery for pituitary adenoma by a surgeon specialized in endoscopic and microscopic surgery, preferring surgery over drug therapy due to intolerance of side effects and a strong desire for surgical intervention, with complete clinical data available. Tumor size was determined based on the maximum anterior, cranial, or transverse diameter, categorizing tumors as microadenomas (≤10 mm) or macroadenomas (>10 mm, <30 mm). Suprasellar extension of large adenomas was graded using Hardy grading, with Knosp scores of 3 and 4 indicating cavernous sinus invasion. Combined imaging and pathology (Knosp grade < 3, postoperative pathology Ki67 < 3%) with intraoperative observation confirmed the diagnosis of non-invasive pituitary microadenoma and macroadenoma.

#### 2.1.3 Case exclusion criteria

Exclusion criteria involved patients with coagulation disorders and malignant tumors, recent treatments with chemoradiotherapy or gamma knife, incomplete case data, recurrent pituitary adenomas or multiple nasal and sinus surgeries, and non-pituitary adenomas in the sellar region such as Rathke cleft cysts or craniopharyngiomas.

### 2.2 Method

#### 2.2.1 Preoperative preparation

Preoperative preparation entailed a general physical examination, evaluation of the tumor’s shape and location, and anatomy of the sphenoid sinus and nasal cavity using pituitary magnetic resonance imaging (MRI) and three-dimensional computed tomography (CT). Preoperative hormone levels and internal environment were assessed, and abnormalities were treated until surgical needs were met. Atypical MRI cases underwent computed tomography angiography (CTA) for aneurysm exclusion. Nasal irrigation was performed for 3 days prior to surgery, nasal hair was trimmed, and prophylactic antibiotics were administered half an hour before surgery. Postoperative care included daily nasal irrigation with physiological saline.

#### 2.2.2 Operative method

For the operative method, the same surgical teams, experienced in pituitary tumors and specializing in endoscopy and microscopic surgery, respectively, performed the procedures. Endotracheal intubation and general anesthesia were used, along with a Storz endoscope, supporting light source, imaging system, and endoscopic surgical equipment. To mitigate random factors like individual interpretation differences, patients admitted from June 2019 to June 2021 were treated using a microscope, while subsequent patients received endoscopic treatment. A nasal septum mucosal flap was utilized in all endoscopic surgeries, and tumors with pseudocapsule underwent extracapsular resection where feasible.

Microscope group: After administering general anesthesia, the patient’s head was tilted back 15° in a supine position and fixed on a head frame. Routine disinfection and sterile sheet laying were performed, followed by nasal mucosa contraction using epinephrine and saline cotton via a right single-nostril approach. After nasal dilatation, the septal nasal mucosa was incised, and the nasal speculum was inserted. The anterior wall of the sphenoid sinus was opened, a part of the sellar floor removed, and the mucosa of the sellar floor sinus electrocoagulated and resected. Bone fragments eroded by the tumor were curetted, and after incising the dura mater of the sellar floor, the tumor tissue was curetted and aspirated. Cerebrospinal fluid leakage from the sellar diaphragm was monitored, and if present, a conventional fat graft was used. The sella turcica was filled, the nasal mucosa retracted, bleeding stopped, and both nasal cavities filled with Vaseline gauze.

**Figure 1:**
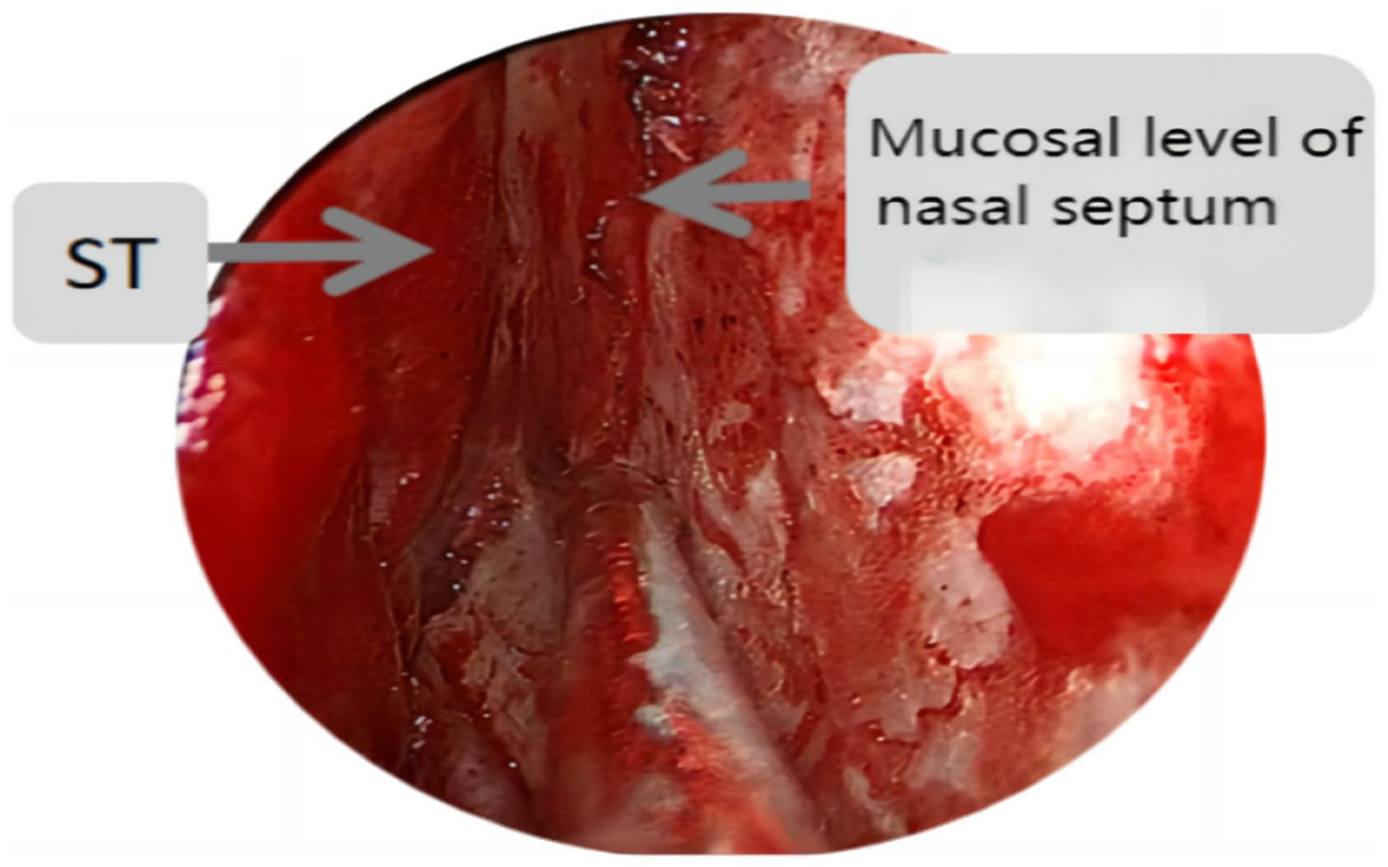
St represents the superior turbinate, this figure shows the incision position of the nasal septal mucosal falp not higher than the level of the ipsilateral superior turbinate.

**Figure 2:**
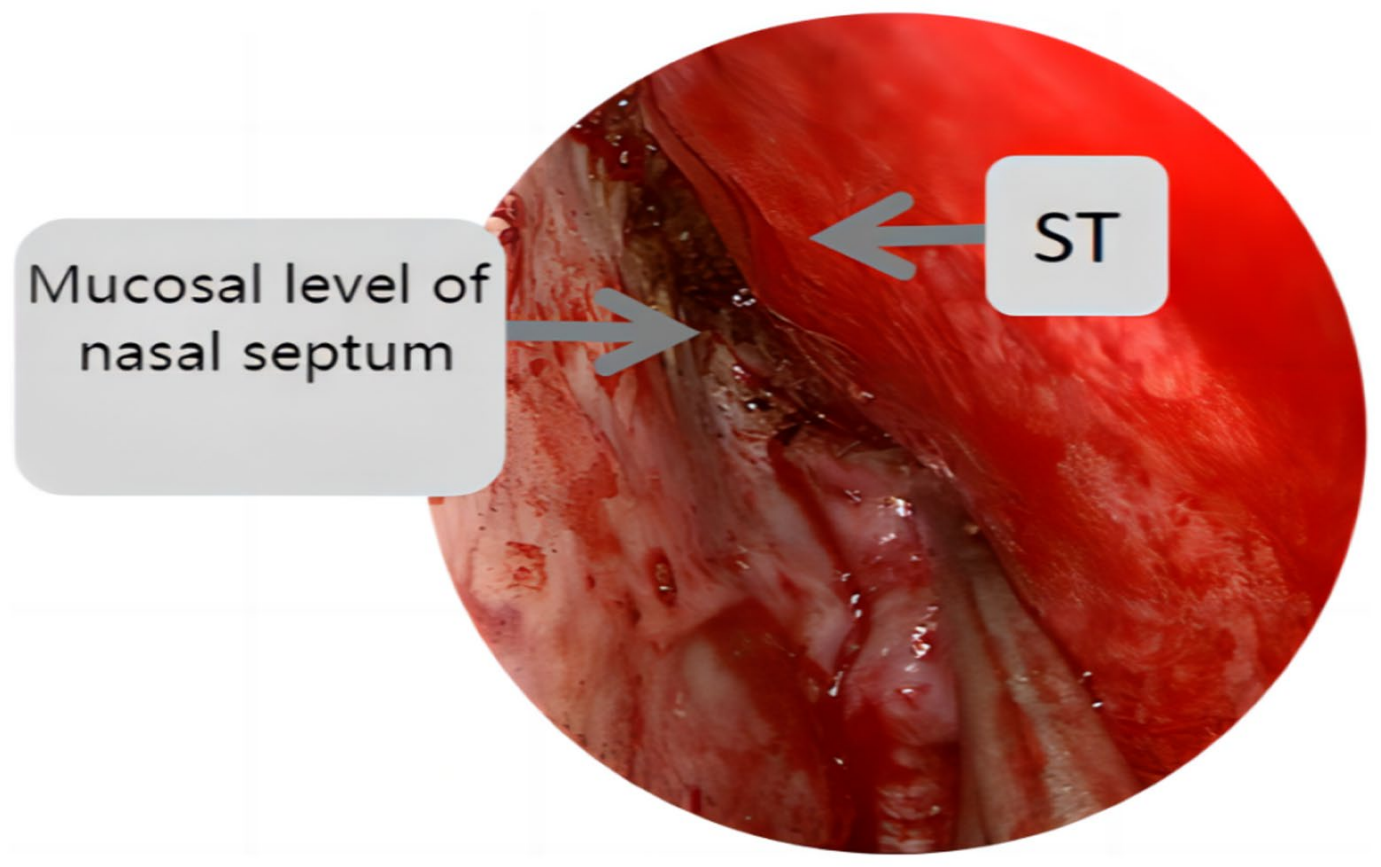
St represents the superior turbinate, this figure shows the incision position of the nasal septal mucosal falp higher than the level of the ipsilateral superior turbinate.

Endoscopic group: General anesthesia was administered, and the patient was placed in the supine position with the head tilted back 15°, fixed using a head ring. Routine disinfection and laying of sterile sheets were performed. The nasal mucosa of the septum was contracted using epinephrine and saline cotton, and incised based on a double-nostril approach for endoscopy. The incision on the superior edge of the nasal septum mucosal flap was not to exceed the level of the ipsilateral superior turbinate for adequate intraoperative exposure, as depicted in Figure 1. In cases of difficult intraoperative exposure and surgical operation, the preservation of the olfactory mucosal flap was not chosen, as shown in Figure 2. The incision began at the lower edge of the sphenoid sinus opening and progressed horizontally along the nasal septum mucosa, turning downward vertically at the level of the lower edge of the middle turbinate and then backward, preserving the main sphenopalatine artery at the root of the mucosal flap for blood supply. A portion of the perpendicular plate of the ethmoid bone was removed, the anterior wall of the sphenoid sinus opened, and the sinus septum cleared. The sellar floor was exposed and removed to form a bone window. The dura mater of the sellar floor was incised, and the tumor tissue removed in parts. The tumor was then separated and removed, achieving hemostasis in the tumor cavity. Pituitary adenomas with a pseudo capsule were resected outside the pseudo capsule as far as possible. During the operation, any instances of CSF leakage were checked, the sellar septum repaired with artificial dura mater, the sellar filled with a gelatin sponge, the sellar floor supported with hard artificial dura mater, and glued with fibrin. After achieving hemostasis of the nasal mucosa, both nasal cavities were filled with an expansion sponge.

#### 2.2.3 Tumor removal rate and preoperative symptom improvement rate

Follow-up examinations were conducted for all patients using computed tomography (CT) and MRI of the sphenoid sinus, along with assessments of visual acuity, field of view, and hormone levels. The recovery rate of vision, visual field, hormone levels, and tumor resection rate were recorded in detail, and the examinations were rechecked after 3 months for verification.

#### 2.2.4 Complication rate

We conducted olfactory testing and observed the recovery of olfactory function for all endoscopic patients through outpatient follow-up before surgery and 3 months later. Observation during hospitalization and follow-up through outpatient follow-up was conducted to determine the incidence of cerebrospinal fluid rhinorrhea, the rate of patients seeking medical attention again due to nosebleed after discharge, the rate of diabetes insipidus, and whether intracranial infection occurred.

Olfactory detection methods are mainly of two types, subjective and objective. While evaluating subjective olfaction detection methods, there might be subjective differences in the olfaction of patients and fluctuations in the results. Many types of olfactory detection methods are available, and the results of each olfactory detection method vary. Objective olfactory detection directly detects physiological changes in the human body after olfaction; thus, this method partially avoids subjectivity. However, the equipment required is expensive and difficult to use in clinical research. Alobid et al. (15) reported the differences between subjective and objective olfactory detection. We developed and scored a set of olfactory test methods to determine the olfactory condition 3 months after the operation.

Olfactometry of five flavors: Olfactometry Solution: A, acetic acid; B, amyl acetate; C, menthol; D, eugenol; E, 3-methyl indole. Each solution was diluted 10 times, and five solutions were prepared at low-to-high concentrations (labeled 1–5). The test solution was placed approximately 1 cm in front of the patient’s nostrils, and they were asked to sniff two to three times. Using this method, we tested whether the patient could smell a solution relative to the previous solution. Five concentrations (labeled 1–5) and solutions (A to E) were tested, starting from the lowest concentration of 1. During each test, the patients were instructed to breathe fresh air several times to avoid olfactory fatigue. The overall process lasted for approximately 3–5 min. The detection thresholds of the five flavored olfactory test liquids were ≥ 3 in ordinary people. At the end of the test, the patients were scored according to the concentration, and the average value of the five reagents was considered to be the olfactory score. If the patient could not smell the test liquids at any concentration, they were given a score of “0,” and the “0” item was not included in the average calculation. Patients with a score of “0” were included in the olfactory impairment group. Patients who could not smell any solution were included in the group in which olfaction was not restored. Patient olfaction was detected before and 3 months after surgery.

#### 2.2.5 Other relevant perioperative parameters

Perioperative indicators of both groups were recorded in detail according to case information, including operation time, postoperative hospital stay, operation cost, and intraoperative blood loss.

### 2.3 Statistical analysis

Statistical analysis was conducted using SPSS 26 software. Measurement data, such as intraoperative blood loss, operation time, and postoperative hospital stay, were expressed as mean ± standard deviation (x ± s). Enumeration data, including the tumor removal rate, postoperative complication rate, postoperative visual function improvement rate, and hormone level improvement rate of functional pituitary adenomas, were expressed as *n* (%). Statistical significance was considered at *p* < 0.05 (*χ*^2^ test).

## 3 Results

### 3.1 General information

In total, 93 patients who underwent non-invasive pituitary microsurgery through the nasal transsphenoidal approach, endoscopic surgery, and microsurgery for pituitary adenomas between June 2019 and October 2022 at the First Affiliated Hospital of Harbin Medical University were included in this study. They were divided into the neuroendoscopic group (*n* = 58) and the microscopic group (*n* = 35). The endoscopic group had 16 males and 42 females, with a mean age of 50.84 ± 12.65 years. Nine patients had combined visual impairment and visual field defect, and 24 patients had abnormal hormone levels. Among these patients, 29 had nonfunctional adenomas, and 29 had functional adenomas, including two cases of ACTH adenomas, 12 cases of GH adenomas, seven cases of PRL adenomas, four cases of TSH adenomas, and four cases of mixed adenomas. There were 13 patients over the age of 60 years, and 21 patients in this group had underlying diseases.

**Figure 3:**
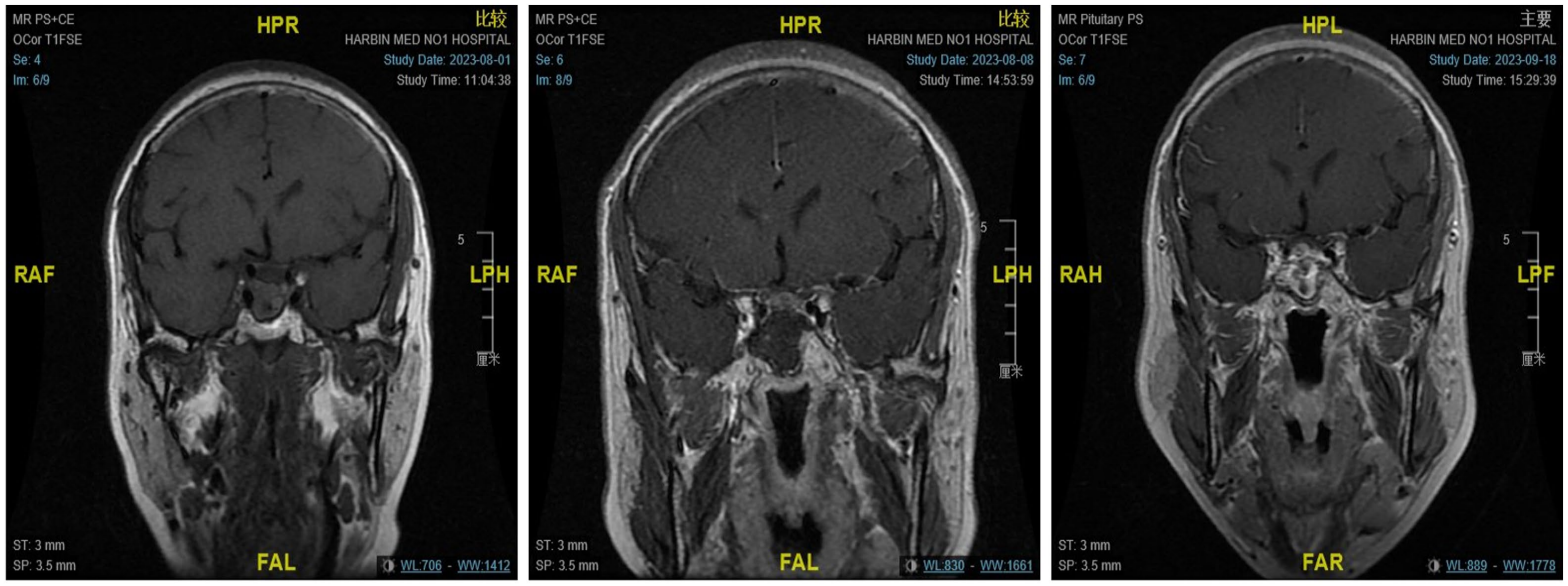
From left to right are the MRI scans of the patients before surgery, on the day after surgery, and 3 months after surgery, respectively.

The microscopic group included 16 males and 19 females, with a mean age of 49.97 ± 12.00 years. Among them, 10 patients had combined visual impairment and visual field defects, and 13 patients had abnormal hormone levels. There were 24 cases of nonfunctional adenomas and 11 cases of functional adenomas, including one case of ACTH adenoma, four cases of GH adenoma, five cases of PRL adenoma, and one case of TSH adenoma. There were eight elderly patients (≥ 60 years old), and seven had underlying diseases. No significant differences were found between the groups. The GTR (GTR is defined as complete tumor resection with no increase in imaging findings) rate of endoscopic surgery was 88.57%, and the STR(STR is defined as a tumor resection degree of over 90%)rate was 11.43%. The GTR rate of microsurgery was 93.10%, and the STR rate was 6.89%. The difference was not statistically significant (*p* > 0. 05; see Table 1; Figure 3).

**Table 1:** General information of patients before surgery.

Project	Microscope group *n* (%)	Endoscopic group *n* (%)	Total	*χ* ^2^	*p*
Preoperative visual acquity and visual field deficits					
Have	25 (71.43)	49 (84.48)	74 (79.57)	2.288	0.130
None	10 (28.57)	9 (15.52)	19 (20.43)		
Preoperative hormone levels were					
Normal	22 (62.86)	34 (58.62)	56 (60.22)	0.704	0.703
Abnormal	13 (37.14)	24 (41.38)	37 (39.77)		
Gender					
Male	16 (45.71)	16 (27.59)	32 (34.41)	3.178	0.075
Female	19 (54.29)	42 (72.41)	61 (65.59)		
Tumor type					
Nonfunctional	22 (62.86)	29 (50.00)	51 (54.84)	1.457	0.227
Functional type	13 (37.14)	29 (50.00)	42 (45.16)		
ACTH type pituitary adenoma	1 (2.86)	1 (1.72)	2 (2.15)	0.133	0.715
GH type pituitary adenoma	4 (11.43)	12 (20.69)	16 (17.20)	1.314	0.252
PRL type pituitary adenoma	5 (14.29)	7 (12.07)	12 (12.90)	0.095	0.757
TSH type pituitary adenoma	1 (2.86)	4 (6.90)	5 (5.38)	0.700	0.403
Mixed type pituitary adenoma	1 (2.86)	4 (6.90)	5 (5.38)	0.700	0.403
GTR	31 (88.57)	54 (93.10)	85 (91.40)		
STR	4 (11.43)	4 (6.89)	8 (8.60)	0.570	0.752
Partial	0	0	0		

### 3.2 Improvement rate of preoperative symptoms

In the microscope group, 10 patients had impaired vision and an abnormal visual field before the operation; nine patients showed complete recovery, and one showed partial recovery after the operation. Hormone levels were abnormal in 13 patients before surgery, and the hormone levels of all patients returned to normal after the operation. In the endoscopic group, nine patients had impaired vision and an abnormal visual field before the operation; eight patients showed complete recovery, and one showed partial recovery after the operation. Hormone levels were abnormal in 24 patients before the operation. The hormone levels returned to normal in 23 patients after the operation, and did not return to normal in one case; the difference was not statistically significant (*p* > 0. 05; see Table 2).

**Table 2:** Comparison of improvement rate of preoperative symptoms between the two groups.

Project	Microscope group *n* (%)	Endoscopic group *n* (%)	Total	*χ* ^2^	*p*
Visual acuity and visual field were completely restored.	9 (90.00)	8 (88.89)	17 (89.47)	0.006	0.937
Visual acuity and visual field were partially restored.	1 (10.00)	1 (11.11)	2 (10.53)		
Hormone levels have not returned to normal.	0 (0.00)	1 (4.17)	1 (2.70)	0.557	0.456
Hormone levels return to normal.	13 (100.00)	23 (95.83)	36 (97.30)		

### 3.3 Postoperative complications

After microsurgery, diabetes insipidus was observed in five patients, presenting mild symptoms. These symptoms improved on the same day following the subcutaneous administration of pituitrin. Additionally, 10 patients experienced damage to the diaphragma sellae during the operation, resulting in intraoperative low-flow CSF leakage. Two patients exhibited persistent postoperative cerebrospinal fluid rhinorrhea, despite packing with hemostatic materials and blocking with a nasal mucosal flap. These cases were resolved through conservative treatment during hospitalization. Furthermore, two instances of infection were managed with physical cooling and corresponding antibiotic treatment, leading to symptom relief within 3 days. After three months, the olfactory function fully recovered in 33 cases and partially in two cases. There were six cases of diabetes insipidus in the endoscopic group, 21 cases of sellar diaphragm damage during the operation, followed by intraoperative low-flow CSF leakage, five cases of persistent postoperative cerebrospinal fluid rhinorrhea after hemostatic material packing was used and nasal mucosal flap occlusion was performed. These issues were cured by conservative treatment during hospitalization. Four cases of infection, one case of epistaxis, and 37 cases of olfactory function recovered after 3 months. There were 13 cases of partial recovery and eight cases of no recovery. All complications were treated in the same manner as in the microscopic group. The recovery rate of olfactory functions in the microscopic group was significantly higher than that in the endoscopic group, and no significant differences were found in the other complications (*p* > 0.05; see Table 3).

**Table 3:** Comparison of the incidence of postoperative complications between the two groups.

Project	Microscope group *n* (%)	Endoscopic group *n* (%)	Total	*χ* ^2^	*p*
Postoperative cerebrospinal fluid leakage	2 (5.71)	5 (8.62)	7 (7.53)	0.265	0.607
Intracranial infection	2 (5.71)	4 (6.90)	6 (6.45)	0.051	0.822
Nosebleed	0 (0.00)	1 (1.72)	1 (1.08)	0.61	0.435
Diabetes insipidus	5 (14.29)	6 (10.34)	11 (11.83)	0.325	0.569
No recovery of olfactory function	0 (0.00)	8 (13.79)	8 (8.60)		
Partial recovery of olfactory function	2 (5.71)	13 (22.41)	15 (16.13)	11.298	0.004
Complete recovery of olfactory function	33 (94.29)	37 (63.79)	70 (75.27)		

### 3.4 Other perioperative relevant parameters

The mean intraoperative blood loss in the microscope group was 19.57 ± 17.29 mL. The average cost of operation was 1,392.12 ± 160.71 dollars. The mean operation time was 2.45 ± 0.47 h. The mean postoperative hospital stay was 7.23 ± 1.83 days. The average tumor diameter was 18.09 ± 6.42 mm. The mean intraoperative blood loss in the endoscopic group was 32.07 ± 28.78 mL. The average cost of operation was 3,513.79 ± 469.40 dollars. The mean operation time was 3.93 ± 1.44 h. The mean postoperative hospital stay was 6.02 ± 2.78 days. The average tumor diameter was 24.50 ± 3.12 mm. The intraoperative blood loss, operation time, and operation cost in the microscope group were less than those in the endoscopy group. The postoperative hospital stay in the microscope group was significantly longer than that in the endoscopy group (*p* < 0. 05). There was significant difference in the average tumor diameter between the two groups (*p*<0.05; Table 4).

**Table 4:** Comparison of other perioperative-related indexes between the two group.

Project	Microscope group *n* = 35	Endoscopic group *n* = 58	*t*	*p*
Intraoperative blood loss (ml)	19.57 ± 17.29	32.07 ± 28.78	−2.325	0.022
Time of operation (h)	2.45 ± 0.47	3.93 ± 1.44	−7.209	0.000
Postoperative hospital stay (d)	7.23 ± 1.83	6.02 ± 2.78	2.291	0.024
Tumor diameter (mm)	18.09 ± 6.42	24.50 ± 3.12	−5.531	0.000
Average cost of surgery ($)	1392.12 ± 160.71	3513.79 ± 469.40	−31.499	0.000

### 3.5 Factors affecting recovery of olfaction in the endoscopy group

No significant difference in olfaction before surgery and 3 months after surgery was found in the group with complete olfaction recovery. However, significant differences in olfaction before and 3 months after surgery were observed between the groups with partial and no olfaction recovery (*p* < 0.001; Table 5).

**Table 5:** Olfaction of 58 patients before and after the operation.

	Olfactory complete recovery group (*n* = 37)	Partial recovery of olfaction group (*n* = 13)	Unrestored olfaction group (*n* = 8)
Preoperative olfaction	4.62 ± 0.49	4.769 ± 0.44	4.625 ± 0.52
Postoperative olfaction 3 months	4.54 ± 0.51	1.692 ± 0.48	0
*t*	1.782	17.321	25.276
*p*	0.083	0.000	0.000

Univariate analysis results showed that sex, age, intraoperative superior turbinectomy, middle turbinectomy, preoperative pituitary function, postoperative electrolyte disturbance, pathological type, and pituitary adenoma with stroke were not significant factors (*p* > 0.05), while the position of the nasal septum mucosal flap incision was significant (*p* < 0.05; Table 6).

**Table 6:** Results of univariate analysis of hyposmia 3 months after endoscopic transsphenoidal surgery for pituitary adenoma.

Clinical factors	Unrestored olfaction group (*n* = 8)	Olfactory complete recovery group (*n* = 37)	Partial recovery of olfaction group (*n* = 13)	*p* value
Gender (female/male, example)				
Male	2	10	4	0.720
Female	6	27	9	0.655
Age $\left(\bar{x}\pm s\right)$	46 ± 10.84	51.30 ± 17.55	49.15 ± 18.34	
Superior turbinectomy				
Have	1	2	1	
None	7	35	12	0.484
Middle turbinectomy				
Have	1	0	0	
None	7	37	13	0.069
Position of incision above nasal septum mucosal flap				
Above the ipsilateral superior turbinate	7	9	4	
Below the ipsilateral superior turbinate	1	28	9	0.007
Preoperative pituitary function				
Abnormal	6	20	8	
Normal	2	17	5	0.605
Electrolyte disturbance after operation				
Have	2	13	4	
None	6	24	9	0.550
Pathological type				
Functional type	4	15	6	
Nonfunctional type	4	22	7	0.966
Pituitary tumor stroke				
Have	1	3	2	
None	7	34	11	0.724

Logistic regression analysis was performed if the result of the univariate analysis was statistically significant. It was conducted to assess the risk factors for hyposmia 3 months after pituitary adenoma surgery (*n* = 58), with the position of the nasal septal mucosal flap incision (OR = 3.50, *p* = 0.015, 95% CI: −2.261–0.244) identified as a risk factor (Table 7).

**Table 7:** Analysis of hyposmia 3 months after endoscopic transsphenoidal surgery for pituitary adenoma.

Risk factors	Wald	*P* value	OR value	95%CI
Position of nasal septal mucosal flap incision	5.929	0.015	3.50	−2.261 ~ −0.244

## 4 Discussion

### 4.1 Surgical procedure for pituitary adenoma

By comparing published studies on the two surgical techniques over the past decade, the advantages and disadvantages of the two techniques can be summarized as follows: endoscopy can provide a wider field of view than microscopy. Combined with angle microscopy, it can help visualize the suprasellar, parasellar, and infrasellar regions, and can also remove sellar nodules to enter the anterior cranial fossa. It can be used to widely expose and directly explore the cavernous sinus. Also, the advantage of removing the clivus bone to remove invasive tumors is widely used in TS surgery (16–18). Tumors that previously required craniotomy, especially those that could be removed only partially or could not be surgically removed at all, can now be removed via endoscopy. However, the disadvantage of this technique is that endoscopes and surgical instruments may limit their operational capabilities in limited space, requiring the assistance of another surgeon to operate the lens (19). Also, the lens is prone to blood contamination, requiring frequent extraction and cleaning (13, 20).

The microscope has a three-dimensional view and precise microanatomy, which is more convenient for a single-person operation (13, 20–22); however, this approach only places the endoscope through one nostril, further limiting the field of view of the saddle. Owing to the limitations of the visual field caused by endoscopy, the removal of large adenomas usually relies on tactile feedback from instruments, especially on the sellar side. The tumor may be adjacent to the medial cavernous sinus or may invade it, making it difficult to achieve complete resection within a limited visual field and increasing the chances of tumor recurrence (17).

Owing to the limitations of the instruments, researchers have not been able to combine the advantages of the two techniques, and thus, they are usually limited to using endoscopes to observe whether there are residual tumors after tumor resection under a microscope.

Most surgeons specializing in pituitary surgery agree that endoscopic surgery has few advantages over microscopic surgery, and might have some disadvantages when used for removing smaller tumors (23). However, studies supporting this view are lacking. In clinical practice, we found that transsphenoidal surgery for non-aggressive pituitary microadenomas and macroadenomas has a higher removal rate, requires shorter operation time, and leads to a lower incidence of postoperative complications after curettage of the tumor boundary under the microscope. Therefore, we performed a comparative endoscopic and microscopic study of these tumors, and the results are discussed below.

### 4.2 Tumor removal rate and improvement of preoperative symptoms

The endocrine response and tumor removal rates are key indicators of functional pituitary adenomas. Some studies have shown that endoscopic surgery may result in a better endocrine cure rate (24). This conclusion is particularly applicable to patients with growth hormone-secreting adenomas, in which tumors invading the cavernous sinus are more effectively resected using endoscopic techniques. Meanwhile, research has pointed out that experienced surgeons using microscopes to remove microadenomas that cause Cushing’s disease (CD) are particularly effective (25). This is because endoscopy, which has a broader field of view than microscopy, can remove tissue spaces that are not visible under the microscope, including tumors that invade the cavernous sinus. However, no significant difference was observed in the rate of hormonal improvement between the endoscopy and microscopy groups. The participants in this study had non-invasive pituitary microadenomas and macroadenomas. The tumors had a small diameter; non-invasive tumors rarely grow into the cavernous sinus and other tissue spaces. Microscopic curettage can scrape the tumor boundary to achieve a higher GTR rate. In this study, 24 patients in the endoscopic group and 13 patients in the microscopic group had preoperative hormonal abnormalities; thus, the sample size was small. The findings of two large single-center studies that used endoscopic nasal approaches were compared, and the results showed that endoscopic surgery is at least as effective as similar microscopic approaches in achieving endocrine remission rates in functional adenomas, with similar or fewer complication rates (2, 26). Although these comparisons have many limitations, the most notable are the use of historical controls, comparison of tumors that have dissimilar size or aggressiveness, incidence of complications, and inclusion of various types of endoscopic approaches. Other smaller single-center studies compared the experiences of these two methods. They concluded that the endoscopic TS method was safe and effective with fewer postoperative complications than the microscopic method (3, 27). However, some studies found the opposite pattern. In a smaller retrospective study on 137 patients with Cushing’s disease, surgical complications, hormonal outcomes, and early remission rates (86% vs. 83%) were comparable between microscopic and endoscopic surgeries. However, the recurrence rate within 10 years was lower in patients who underwent microscopic surgery, especially in those with large adenomas (28). In the comparison of functional pituitary adenomas, the results of different centers were different, their conclusions were contradictory, and the conclusions of pituitary adenomas of different hormone types were also dissimilar; therefore, further studies are needed to provide stronger evidence for drawing definite conclusions.

Chen et al. performed a meta-analysis of all available global data from 1999 to 2020 in a large-scale study (29). They compared the results of endoscopic and microscopic transsphenoidal surgery for pituitary adenomas. In 29 retrospective case–control studies that included 7,774 patients with functional and nonfunctional pituitary adenomas, no significant difference was found in the rate of resection, remission of postoperative hormone secretion, or overall incidence of complications. Endoscopic surgery is less likely to be performed than microscopic surgery to avoid postoperative diabetes insipidus. When the sample size was sufficiently large, no significant difference was found in the tumor removal rate between endoscopy and microscopy, which was consistent with the conclusions of this study. However, a more accurate conclusion needs to be provided by large-scale studies.

### 4.3 Postoperative complications

Postoperative changes in the secretion of antidiuretic hormone may cause fluid and electrolyte disturbances, which are common in the clinical setting. One reason might be that the traction of the pituitary stalk caused by iatrogenic manipulation leads to trauma and mechanical interference with the release of the ADH in reserve. The incidence of DI ranged from 0.3 to 45%. However, this percentage may vary owing to inconsistent definitions of each type of report, different follow-up intervals, and the experience of the surgical teams (30, 31). The DI rates in this study were 14% for the microscopic group and 10% for the endoscopy group (*p* > 0.05), with no significant difference in incidence.

The leakage of CSF is a common complication of sphenoid sinus surgery and can be avoided by taking adequate measures to reduce injury to the sellar diaphragm. Additionally, CSF leakage is closely associated with intracranial infections. Bacterial pathogens may enter the intracranial cavity if not treated promptly, leading to severe consequences. Multiple meta-analyses and studies (32, 33) have shown that the CSF leakage and infection rates of endoscopy were lower than those of microscopy, but no such difference was found between the two techniques in this study. However, in a study, Chen et al. (34) analyzed the medical records of 239 patients with giant adenomas, including 168 cases of endoscopic transsphenoidal surgery and 71 cases of microscopic transsphenoidal surgery, and showed that the neuroendoscopic group had a significantly lower rate of postoperative complications, including diabetes insipidus, intracranial infection, and CSF leakage, than the microscopic group. The size of the tumor samples significantly affected the results. The patients selected in this study had non-invasive pituitary macroadenoma and microadenoma, which have a lower probability of damaging the sellar diaphragm and causing postoperative CSF rhinorrhea.

### 4.4 Other perioperative relevant parameters

In this study, intraoperative blood loss, operation time, and operation cost were significantly lower in the microscope group than in the endoscopic group, which occurred probably because pedicle nasal septum mucosal flap reconstruction was implemented in the endoscopic TS group to reduce the risk of postoperative CSF leakage (35, 36). When the pedicle mucosal flap is prepared, the sphenoid sinus and the mucosal blood vessels are easily damaged, and the time and cost of endoscopic surgery increase. And endoscopic surgery has higher requirements on the three-dimensional sense of the space of the operator and the assistant, that the endoscopic lens is easily polluted by blood in the operation area during the operation. The lens must be repeatedly cleaned to improve the efficiency of the operation. The proficiency of the operator and the assistant also significantly affects the progress of the operation; microscopic surgery does not have these limitations. Little et al. compared the surgical outcomes of the application of pure microscopy and pure endoscopic transsphenoidal surgical methods for the treatment of non-functional pituitary adenomas (13). In total, 82 patients underwent microscopic surgery and 177 patients underwent endoscopic surgery. No significant differences were found in the overall resection rate (> 80% for both groups), mean extent of resection (98% for both groups), length of hospital stay, rate of unplanned readmission, or surgical complication rate between the groups. Although the operation time was significantly shorter in the microscopic group, a significantly greater proportion of patients in the trial had new hypoplasia after 6 months (28.4% vs. 9.7%). However, in Rotenberg’s study (14), the operation time using an endoscopic method was significantly shorter than that of the microscope method, which might be caused by the different times, learning curves, and proficiency of endoscopic TS operators in different surgical teams. This difference may improve with advancements in endoscopic imaging technology.

In this study, it was observed that the tumor size in the endoscopic group was significantly larger than that in the microscope group. This was attributed to the initial stage of the experimental design, where larger adenoma patients were deliberately selected for inclusion to avoid errors caused by the unnecessary production of mucosal flaps for some adenomas. Consequently, a uniform approach to nasal mucosal flap production was adopted during surgery. Morten’s study (37) suggested that, for optimal outcomes, endoscopic resection should be predominantly applied to adenomas larger than 2 cm. Similarly, other studies (38, 39) concurred with these findings, demonstrating that the difference in tumor size did not influence the experimental results when a sufficiently large sample size was utilized.

In a Mayo Center study on surgical cost (38), the researchers reported that the operation cost of the microscope group was 16% lower than that of the endoscope group. The operation time was 48 min shorter than that in the endoscope group (*p* < 0.05), which was similar to the findings of this study. However, no significant differences in the length of hospital stay were found. The cost of endoscopy is higher than that of microscopy, and the increase in the use of intraoperative hemostatic materials and operation time further increases the cost. During this study, most patients in the microscopy group were diagnosed and treated between 2020 and 2021, which was the period of the COVID-19 pandemic. The bed turnover rate decreased and the length of hospital stay increased because of the pandemic, which partly affected the experimental results.

### 4.5 Factors affecting recovery of olfaction in the endoscopy group

Preparation and peeling of the mucosal flap: In the endoscopic treatment, the double-nostril approach was mainly used. Although, generally, the operation for non-invasive pituitary microadenomas and macroadenomas is endoscopic single-nostril rather than double-nostril, we found in the diagnosis and treatment that the operation time of the single-nostril approach is longer than that of the double-nostril approach; additionally, the intraoperative blood loss is greater in the single-nostril approach than in the double-nostril approach. In the double-nostril approach, when two medical personnel perform the surgery, the operation space can be increased, and the cooperation and coordination during the operation can be improved, which can greatly enhance the operation efficiency. Zeng et al. (40, 41) reported that both single and dual-nostril approaches are effective in removing pituitary adenomas, and they found no significant difference in operation time and postoperative complications.

A Hadad mucosal flap (42) was used in this study. In a meta-analysis of 29 studies conducted over 20 years by Zamanipoor et al. (43), the researchers found that this valve-making method can effectively reduce the chances of postoperative CSF leakage. Postoperative cerebrospinal fluid leakage has decreased significantly in recent years, probably because of the development and improvement of new closure techniques, such as the Hadad-Bassagasteguy flap and gasket seal. Meanwhile, the average diameter of the tumor in the endoscopic group in this study was 24.50 ± 3.12 mm (see Table 5). Study (44) have shown that when the pituitary adenoma is larger than 2.5 cm in diameter, the use of Hadad mucosal flap can achieve good results.

The change in the anatomy of the nasal cavity is the main factor leading to nasal symptoms after surgery (45), and injury to the olfactory mucosa is a key reason for hyposmia. Escada et al. (7) described the location of olfactory neurons in the superior and middle turbinates and their corresponding septal mucosa within about 1.5 cm^2^ of the skull base. Gomez et al. (7) also conducted autopsy studies and found that olfactory neurons are widely distributed in the nasal mucosa. They are highly concentrated in a small area of the upper turbinate (1 cm^2^) and on top of the nasal cavity.

However, in previous studies (39, 42, 46, 47), many authors mentioned that the upper edge of the incision is 1–2 cm from the top of the nasal cavity, without specifying a method to avoid mucosal damage in the olfactory region while making the nasal septum mucosal flap incision. This has led to a slight variation in the surgical treatment of pituitary tumors. According to a study (7), the midpoint of the lower edge of the upper turbinate to the top of the nasal cavity is approximately 1.5 cm. We speculated that making an incision at the nasal septum mucosal flap that is lower than the level of the ipsilateral upper turbinate might effectively protect the sense of smell.

All patients included in this study were informed of the detailed steps of endoscopic transsphenoidal surgery before they underwent surgery. All patients were initially supposed to retain the nasal septum mucosa on the same side corresponding to the superior turbinate; however, if the progress of the operation was hindered, the plan was changed to make the procedure safe for the patients to the greatest extent. Additionally, the nasal septal mucosa corresponding to the ipsilateral superior turbinate can protect the olfactory area (7), as reported in other studies, It can also indicate that preserving the olfactory mucosa is helpful in restoring postoperative olfactory function.

Few studies have investigated factors related to hyposmia. When the patients in this study were followed up, 58 of them were found to have profuse nasal secretions, and only six patients could smell the high concentration of reagent solution within 1 week after the operation. Nasal secretion and exudation decreased in most patients approximately 1 month after the operation. At that time, patients could smell strong odors in the environment. After 3 months, the sense of smell of the patients was stable. It takes approximately 6 weeks to repair nasal mucosa injury after surgery, and approximately 3 months to restore the structure and function of the cilia on the surface of olfactory cells (48); therefore, 3 months after surgery was selected as the node of olfactory detection.

In this study, we did not consider the pathological classification of pituitary adenomas as risk factors for postoperative dysosmia. However, Actor et al. and Hart et al. (8, 9) found that the olfaction of patients with growth hormone-induced pituitary adenoma improved after surgery, which occurred probably because growth hormones can cause nasal mucosal hypertrophy and turbinate hyperplasia to cover the olfactory fissure area. This can increase the difficulty in gas circulation. In contrast, the nasal mucosa and turbinate returned to normal after surgery.

In this study, although the size of the pituitary adenoma and the level of preoperative pituitary function were not influencing factors of postoperative hyposmia in the statistical analysis, they might be related to hyposmia after the analysis. The larger the size of the pituitary adenoma, the more disordered the anatomical structure and surrounding tissue, and the more delicate the intraoperative procedure. Rotenberg et al. (10) found that functional tumors were smaller. However, the degree of bone fragility and mucosal hypertrophy was higher than that of nonfunctional pituitary adenomas, which increased the effect on surrounding tissues and the chances of damage to the olfactory mucosa during surgery.

The development of transsphenoidal surgery, improved the overall quality of life while reducing surgical complications. In this study, no significant difference was found in the incidence of complications between the two groups. However, the recovery rate of olfactory function was significantly lower in the endoscopic group compared to that in the microscopic group. This occurred probably because endoscopic transsphenoidal surgery often sacrifices a portion of the middle/superior turbinate and abandons unilateral septal mucosa to preserve the working space for forceps and equipment. It also sacrifices the posterior ethmoid sinus to access the sphenoid sinus through a single nasal cavity. Damage to the nasal cavity is relatively extensive; patients often experience abnormal olfactory function after surgery. Recovery takes a long time, and the olfactory function may be lost permanently; in severe cases, it can even affect the sense of taste (49, 50). There is also a higher incidence of delayed epistaxis (51).

Destruction of the nasal anatomy due to smaller microadenomas is costly. Effective measures must be taken to reduce the damage to the nasal cavity. For example, intraoperative sinus dissection and periodic endoscopic debridement, examination, and irrigation are required to reduce chronic mucosal inflammation and recurrent infection due to the narrow natural drainage pathway of the sinuses. Proper treatment can prevent or decrease the occurrence of mucocele (52, 53). In this study, all six patients with intracranial infection developed postoperative olfactory dysfunction; however, no study has shown a correlation between intracranial infection and postoperative olfactory dysfunction. All patients in the endoscopic surgery group applied nasal washers for nasal flushing after surgery. This practice reduced the chances of chronic mucosal inflammation or infection affecting olfactory function to some extent, although it cannot be ruled out that patients may have stopped using them after they were discharged from the hospital.

Two other factors might have affected the sense of smell after the operation. First, the overall follow-up time in this study was 3 months, and the olfaction of some patients may have improved over time. However, no study has reported on the specific time required for recovery. Second, during the follow-up, we found that many patients had more nasal secretions and scabs, which led to poor nasal ventilation and clogged the nasal cavity. Almeida et al. (54) showed that postoperative nasal scabbing and secretion can prevent air from passing through the olfactory area and affect the sense of smell. Regular nasal irrigation and endoscopic debridement can clean the nasal cavity and reduce inflammatory stimulation, which may promote the recovery of olfaction (55).

The superior and middle turbinates were preserved during the operation. Only four patients with problematic exposure to the surgical field underwent superior turbinectomy, and one patient underwent middle turbinectomy. Mariano et al. (11) found that middle turbinectomy may increase nasal ventilation and airflow in the superior nasal meatus to improve olfaction, but it may also lead to changes in the airflow pattern in the nasal cavity. In this study, superior turbinectomy was performed following the method described by Li et al. (12), in which the lower one-third of the superior turbinate was removed to preserve olfaction without interfering with surgical exposure. Our results were similar to those reported by Li et al., who found that superior turbinectomy was not associated with postoperative hyposmia (12). The treatment of the superior turbinate is also known as lateralization of the superior turbinate. In this procedure, the superior turbinate is pushed to the lateral wall, which increases the exposure of the operation area, and avoids resection of the superior turbinate; however, it may lead to changes in local anatomy and airflow dynamics. Li et al. (12) found no significant difference in olfaction after superior turbinectomy and lateralization. Say et al. (56) found that around 12% of the olfactory mucosa is located on the surface of the superior turbinate. Escada et al. (7) found a small number of olfactory filaments on the surface of the middle turbinate. Although no relationship was found between hyposmia and superior and middle turbinectomy, reducing the damage to the turbinate helps protect more olfactory epithelial cells.

Future studies should also include other methods of mucosal flap production. Rivera et al. (46) proposed an improved approach to produce the nasal septal mucosal flap. An incision is made from the sphenoid sinus opening to the corresponding mucosa of the middle turbinate, on the surgical side of the nasal septum. During the surgery, it is determined whether to expand the mucosal flap incision based on the presence or absence of cerebrospinal fluid leakage, Theoretically, compared to traditional Hadad mucosal flaps, it can cause less damage to the mucosa in the nasal cavity. Upadhyay et al. (45) reported that the olfactory function of patients who underwent bilateral nasal septum salvage mucosal flap surgery was restored to preoperative levels 3 months after surgery. However, the rescue mucosal flap technique cannot completely prevent olfactory decline. Hong et al. (39) and Sowerby et al. (49) found that the olfactory score 6 months after rescue mucosal flap surgery was lower than that before surgery. Additionally, postoperative anatomical changes and intraoperative burning to stop bleeding may affect blood supply to the nasal mucosa after surgery (47).

In this study, we found that the position of the nasal septal mucosal flap incision is a risk factor for the loss of olfactory function 3 months after pituitary adenoma surgery. Among the eight patients whose olfactory function did not recover, seven had incisions above the nasal septal mucosa of the upper turbinate. This finding confirmed our hypothesis that preserving the ipsilateral nasal septal mucosa corresponding to the upper turbinate is sufficient to cover the previously defined olfactory area (7), which can facilitate postoperative olfactory recovery and maximize the safety of the patients.

To summarize, in this study, the position of the nasal septum mucosal flap incision was a risk factor for hyposmia 3 months after the operation. However, the patients’ sex, age, intraoperative superior and middle turbinectomy, preoperative pituitary function, tumor size, postoperative electrolyte disorder, pathological type, tumor apoplexy, or recurrence of pituitary adenoma were not significant risk factors. The lack of statistical significance might be due to the small sample size. The algorithm might have excluded a small number of positive cases of some indicators in the dataset. Thus, future studies need to include a large number of multicenter cases to support the findings of this study.

## 5 Conclusion

Tumor removal and postoperative hormone remission rates were similar between the two groups. In contrast, the microscopic group had a shorter operation time, less intraoperative blood loss, faster olfactory function recovery, and a lower average operation cost. The position of the nasal septal mucosal flap incision was a risk factor for hyposmia at 3 months postoperatively. Hyposmia is less likely to occur when the superior edge of the nasal septal mucosal flap incision is not higher than the lower edge of the ipsilateral superior turbinate.

## Data availability statement

The raw data supporting the conclusions of this article will be made available by the authors, without undue reservation.

## Ethics statement

The studies involving humans were approved by the Ethics Committee of the First Affiliated Hospital of Harbin Medical University. The studies were conducted in accordance with the local legislation and institutional requirements. The participants provided their written informed consent to participate in this study. Written informed consent was obtained from the individual(s), and minor(s)’ legal guardian/next of kin, for the publication of any potentially identifiable images or data included in this article.

## Author contributions

FK: Conceptualization, Data curation, Formal analysis, Funding acquisition, Investigation, Methodology, Project administration, Resources, Software, Supervision, Validation, Visualization, Writing – original draft, Writing – review & editing. WC: Writing – review & editing. QZ: Supervision, Writing – review & editing.
